# Antifouling Potential of Ethyl Acetate Extract of Marine Bacteria *Pseudomonas aeruginosa* Strain RLimb

**DOI:** 10.3390/life13030802

**Published:** 2023-03-15

**Authors:** Nurul Najihah Rawi, Mujahidah Mohd Ramzi, Nor Izzati Abd Rahman, Fazilah Ariffin, Jasnizat Saidin, Kesaven Bhubalan, Noor Wini Mazlan, Nor Atikah Mohd Zin, Julius Yong Fu Siong, Kamariah Bakar, Ahmad Khusairi Azemi, Noraznawati Ismail

**Affiliations:** 1 Institute of Marine Biotechnology, Universiti Malaysia Terengganu, Kuala Terengganu 21030, Terengganu, Malaysiaijaxzt@umt.edu.my (J.S.); kesaven@umt.edu.my (K.B.); atikah@umt.edu.my (N.A.M.Z.); yongjulius@umt.edu.my (J.Y.F.S.); kamariah@umt.edu.my (K.B.); 2 Faculty of Science and Marine Environment, Universiti Malaysia Terengganu, Kuala Terengganu 21030, Terengganu, Malaysia; fazilah@umt.edu.my (F.A.); noorwini@umt.edu.my (N.W.M.)

**Keywords:** antifouling, antibacterial, marine bacteria, *Pseudomonas aeruginosa*, biofilm

## Abstract

Biofouling is defined as the excessive colonization process of epibiotic organisms, ranging from microfoulers to macrofoulers, on any submerged surface in water. Previous research has attempted to explore the antifouling activity of bacterial isolates due to the biofouling problems occurring worldwide. One solution is to inhibit the early stage of fouling using secondary metabolites produced by marine bacteria. This study aims to determine the antifouling activities of the marine microorganism *P. aeruginosa* and to characterize the bacteria isolated as a potential anti-biofouling agent. The bacterial isolate was cultured and isolated on a media culture. The bacteria culture extract was extracted using ethyl acetate and concentrated prior to the bioassay method. It was screened for antibacterial activities against Gram-positive and Gram-negative bacteria, such as *Bacillus cereus, Streptococcus uberis*, *Pseudomonas* sp., and *Vibrio parahaemolyticus*, using the disk diffusion technique. The extract was investigated to verify its bioactivity in the prevention of biofilm formation following the crystal violet assay and aquarium test. The results indicated the inhibition of activity through biofilm formation, with the highest percentage at 83% of biofilm inhibition at a concentration of 0.1563 mg/mL. The bacterial isolate at a concentration of 5% showed the highest reduction in bacteria colonies in the aquarium test (161.8 × 10^3^ CFU/mL compared to 722.5 × 10^3^ CFU/mL for the blank sample). The bacterial isolate was characterized through phenotypic and genotypic tests for species identification. It was identified as a Gram-stain-negative, aerobic, and long-rod-shaped bacteria, designated as RLimb. Based on the 16S rDNA gene sequencing analysis, RLimb was identified as *Pseudomonas aeruginosa* (accession number: OP522351), exhibiting a similarity of 100% to the described neighbor *P. aeruginosa* strain DSM 50071. These results indicated that these isolated bacteria can potentially be used as a substitute for toxic antifoulants to prevent the formation of microfoulers.

## 1. Introduction

**Figure 1:**
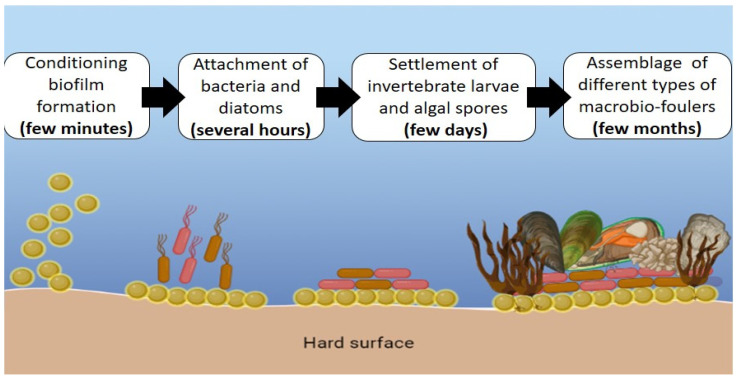
Biofouling formation on submerged surfaces.

In the marine environment, biofouling is a significant problem that affects human activities, especially in the oil and gas industry. Biofouling is defined as a natural process that involves the colonization and excessive growth of epibiotic organisms on submerged surfaces [1]. According to Soliman et al. [2], biofouling is the continuous growth of marine organisms in water. Marine biofouling has been described as a complex process that usually occurs in four distinct phases (Figure 1). In the first phase, as soon as a surface is immersed in seawater, the dissolving of organic molecules—namely carbohydrates, proteins, glycoproteins, and polysaccharides—begins to occur. In a few minutes, these organic molecules create a conditioned biofilm that is vulnerable to fouling organisms [3]. In the second phase, microbial populations of bacteria and diatoms begin to adhere to form the biofilm layer, creating the primary colonizers over the course of several hours. The primary colonizers, predominantly bacteria, act as the dominant components prior to the adherence of macrofouler organisms [4,5]. The surface becomes environmentally suitable, triggering the macrofoulers to attach once biofilm formation is complete. In the third phase, “soft macrofoulers,” such as algae and marine invertebrate larvae, adhere within a few days. The settlement for the entire formation of biofouling is indicated by the presence of marine larvae [6]. In the fourth phase, “hard microfoulers,” including barnacles, mollusks, mussels, and macroalgae, start to grow and inhabit the surfaces. This process occurs over a period of several months [7]. The causes of biofouling can range from microfoulers to macrofoulers, as it occurs due to their undesired accumulation on wet surfaces [8]. Microfoulers comprise marine organisms such as bacteria, phytoplankton, fungi, and diatoms, while macrofoulers consist of limpets, oysters, barnacles, and algae. A previous study reported that the number of fouling organisms comprises approximately 4000 different species, with over 100 species presenting the possibility of damaging the surfaces [9].

The formation of fouling can cause several problems associated with environmental crises and economic loss. The fouler causes an increase in the required materials and costs associated with the maintenance of naval vessels, seawater pipelines, and shipping industries [10]. Furthermore, biofouling can cause pipe-clogging in underwater facilities, which leads to significant increases in maintenance costs [11]. It has been reported that the biofouling issue results in economic losses of over USD 5.7 billion per year across the world [12]. To overcome the formation of biofouling, antifouling paints from various sources have been developed. One solution is using antifouling coating based on organotin compounds, such as tributyltin (TBT) and triphenyltin [13]. These coatings have tin- and copper-based compounds as their active ingredients. These compounds leach into the water through the paint and kill the fouling organisms [2]. While these are efficient antifouling substances, it has been discovered that organotin compounds exert a negative effect on the aquatic ecosystem [14], such as impacting gastropods, causing shell malformation in bivalves, and leading to the depletion of oyster populations [15]. The International Maritime Organization (IMO) introduced resolutions to prohibit the production of TBT antifouling paints from 1 January 2003 onward; furthermore, it banned the use of these paints on ships from 1 January 2008 onward [16].

Following the ban on TBT, alternative methods for preventing biofouling have been suggested. The potential of natural and eco-friendly antifoulants concerns marine organisms, specifically the production of secondary metabolites [17]. Marine organisms can produce secondary metabolites, which show inhibitory activities against biofouling organisms, and can thus result in the development of natural antifoulants [18,19]. As this process already occurs in nature, natural metabolites are considered to be less environmentally damaging than paints containing TBT. Marine microbes can also be beneficial sources of antifouling compounds as they have advantages compared to other marine organisms. Microbes need not be acquired in large quantities from nature as they can be easily cultured and can produce compounds more rapidly [20]. A review study by Liu et al. [21] has demonstrated that about 182 antifouling natural products from marine organisms, algae, and marine invertebrates were reported from August 2014 to May 2020. Amongst these compounds, over half were isolated from marine-derived microorganisms, including 70 compounds from fungi and 31 compounds from bacteria [21]. The *Bacillus licheniformis* strain isolated from *Spongia officinalis* (marine sponge) has been reported to produce exopolysaccharides, which can reduce the development of biofilm for several bacteria [22]. Moreover, Muras et al. [23] proposed that *B. licheniformis* NCTC 10341 can prevent biofilm and soft fouler attachment to non-targeted organisms. Several bioactive compounds in antifouling have been isolated from marine organisms, including corals, sponges, ascidians, bryozoans, and marine microorganisms [24]. As the previous study focused on the microbes associated with a marine organism, the current study has attempted to determine the anti-biofouling activities of *P. aeruginosa*, a marine microorganism isolated from the marine environment. The results can be used to further characterize the bacteria isolated as a potential source of antifouling agents.

## 2. Materials and Methods

### 2.1. Cultivation of Bacteria Isolate

The bacterial isolate was cultured from the culture collections at the Institute of Biotechnology Marine (IMB), Universiti Malaysia Terengganu (UMT), Terengganu, Malaysia. The isolate was inoculated into ZMA (Marine Agar 2216, HiMedia Laboratories LLC, Maharashtra, India) to activate the bacteria from glycerol stock and incubated overnight at 37 °C in the incubator. The pure bacteria colony was isolated via repeated streaking on nutrient-rich (NR) agar plates. Two loops full of pure culture were inoculated into 50 mL of NR broth consisting of the following components, as stated by Azemi et al. [25]: 10 g/L peptone, 10 g/L meat extract, and 2 g/L yeast. It was then incubated at 200 rpm and 30 °C for 12 h.

### 2.2. Biosynthesis of Bioactive Compounds from Bacterial Isolate

The bacteria isolate was inoculated in a 1000 mL Erlenmeyer flask containing a 250 mL working volume of mineral salts medium (MSM)—2.80 g of KH_2_PO_4_, 3.32 g of Na_2_HPO_4_, and 0.25 g of hydrated MgSO_4_·7H_2_O per L—as described by Tamothran et al. [26], with some modifications. This process will increase the production of bioactive compounds from bacteria. The media were supplemented with a nitrogen source, as follows: CO(NH_2_)_2_ at a concentration of 0.25 g/mL and 200 μL of the trace element (TE) per L. The trace element solutions were as follows: dissolved in 0.1 M HCl; FeSO_4_·7H_2_O (2.78 g/L), MnCl_2_·4H_2_O (1.98 g/L), CoSO_4_·7H_2_O (2.81 g/L), CaCl_2_·2H_2_O (1.67 g/L), CuCl_2_·2H_2_O (0.17 g/L), and ZnSO_4_·7H_2_O (0.29 g/L) [27]. This was followed by the addition of 20 g/L of glycerol as a carbon source and 7% (*v/v*) of precultured bacteria. The culture was incubated at 200 rpm and 30 °C for 72 h. The bacterial culture was harvested by centrifuging at 9000 rpm and 4 °C for 5 min using a high-speed refrigerated centrifuge machine (HIMAC CR 22N, Hitachi, Tokyo, Japan). The supernatant was subjected to the extraction method.

### 2.3. Extraction of Bioactive Compounds

The extraction of bioactive compounds from the supernatant was conducted as described by Yin et al. [28]. The pH of the supernatant was measured using a pH meter, and the pH was adjusted to 2.0 with 6 M of HCl. Ethyl acetate was added to the supernatant in the separating funnel at a 1:1 ratio, and the funnel was shaken vigorously. This step was repeated twice, and the upper fraction of the layer was collected and transferred into a clean Erlenmeyer flask. Approximately 20 g of anhydrous sodium sulfate was added to 1 L of the extract to remove the excess moisture. Following this, the solution was concentrated using a rotatory evaporator (BUCHI Malaysia S/B, Selangor, Malaysia) at 40 °C under vacuum pressure. The viscous bacteria culture extract obtained was dissolved in 0.05 M of sodium bicarbonate and adjusted to pH 2.0 using 6 M of HCl. The solution was kept at 4 °C for 24 h to allow acid precipitation to occur, before being centrifuged at 9000 rpm for 5 min. The precipitate obtained was frozen at −80 °C overnight in a biomedical freezer, before being lyophilized using a freezer dry system (Labconco Corporation, Kansas City, MO, USA). The bacteria culture extract obtained was kept for further study.

### 2.4. Antibacterial Assay

For the antibacterial test, the disk diffusion technique [29] was used, with some modifications. The concentrated bacteria culture extract was diluted with absolute methanol to a concentration of 1 g/mL. Subsequently, 30 μL of the sample was pipetted out on the sterile disk and allowed to dry at room temperature. This process was repeated three times. The Gram-positive bacteria, namely *B. cereus* and *S. uberis,* and the Gram-negative bacteria, namely *P. aeruginosa* and *V. parahaemolyticus*, were swabbed on nutrient agar as test strains. All the test strains for this method were revived from the culture collections at the IMB. The dried sample disks were placed on the test strain plate for overnight incubation at room temperature. Standard antibiotic disks (kanamycin (30 μg/disk) and gentamicin (10 μg/disk)) and disks soaked in methanol were used as positive and negative controls. The media plate for this assay was BD DIFCO^TM^ Mueller Hinton agar (MHA) (Becton, Dickinson and Company, Franklin Lakes, NJ, USA). The antibacterial efficacy of a sample against the tested bacteria strains was determined based on the measurement of the inhibition zone (diameter in mm).

### 2.5. Antifouling Test

#### 2.5.1. Crystal Violet Assay

The crystal violet assay as described by Ahmad et al. [30] was used, with some modifications, to study the antifouling properties of the sample toward the biofilm-producing bacteria *P. aeruginosa* in the laboratory test. *P. aeruginosa* was revived from the culture stock at the IMB and used as the test strain for this assay. The colorimetric assay of biofilm formation was stained with crystal violet, which was followed by the removal of unbound staining. Following this, 100 μL of the bacterial culture extract and 100 μL of M63 media were loaded into a 96-well microtiter plate for serial dilution. The dilutions were left enclosed with the lid and incubated for 1 h. After 1 h, 20 μL of the test strain suspension and 130 μL of the media were inoculated into the well of the sample. The wells loaded only with test strain suspension and medium were assigned as a negative control. The biofilm was allowed to develop for 24 h while aerobically incubated at 37 °C. After 24 h of incubation, the plate was decanted, washed, and set to air-dry prior to 15 min of staining with 250 μL of 1% crystal violet at room temperature. The sterile distilled water was used to rinse out the excess stain; this was repeated three times, and it was then left to dry in the oven at 60 °C. After drying, 250 μL of 30% acetic acid, as the solubilizer of the well-bound stain, was pipetted and incubated for 10 min at room temperature. The plate was agitated and measured at OD_595_ using the Multiskan spectrophotometer (ThermoFisher Scientific, Waltham, MA, USA). The percentage of biofilm inhibition was calculated as per the work of Leroy et al. [31]. The entire test was carried out in triplicate.

#### 2.5.2. Aquarium Test

Another antifouling test was conducted in the aquarium at the laboratory to mimic the real environment. The steel panels were cut into square shapes with dimensions of 2.5 cm × 2.5 cm × 0.3 cm. The bacteria culture extract was diluted with a commercial paint thinner that contained a mixture of xylene (CAS No.: 1330-20-7) and ethylbenzene (CAS No.: 100-41-4) as a solvent to dissolve the extracts at a concentration of 5% and 10% of weight per volume [32]. The bacterial culture extracts from Section 2.3 were mixed with blank paint to form a homogeneous paint. The blank paint and commercial reference paint were used as the negative and positive control paint, respectively. The steel panels were painted with an air spray compressor (Model ZL-550Wx2-50L, Uma) to obtain a final dry film thickness of 150 µm for the primer paint and a coating of 100 µm for the sample paint. The panels were left to dry for several days. All the panels were suspended in an aquarium tank containing 141 L of fresh seawater for 3 days, with wave-mimicking conditions. Sterile artificial seawater (1.41 L)—with the following composition as per the work of Chen et al. [33]: NaCl (24.615 g/L), KCl (0.783 g/L), Na_2_SO_4_ (4.105 g/L), MgCl_2_(H_2_O)_6_ (11.06 g/L), and CaCl_2_(H_2_O)_2_ (1.558 g/L)—was added to the seawater to provide the additional essential nutrients. Each panel was retrieved from the aquarium tank at 24 h intervals. The panels were scraped off using a cotton swab and suspended in 1 mL of sterile artificial seawater. This suspension was subjected to serial dilution and spread on ZMA plates. Following this, the agar plates were incubated at 37 °C for the development of bacterial colonies. The colonies were computed using a microbial colony counter (LAPIZ, Medica) [34]. The entire test was performed in triplicate.

### 2.6. Bacterial Isolation and Characterization

#### Morphological Characterization (Gram-Staining Test)

Microscopic observations of the bacteria isolate were performed using the following method, which was described by Ligo et al. [35]. The bacterial isolate was smeared using an inoculation loop and heat-fixed on the glass slide. Slightly heating the slide has been found to help with cell adhesion and prevent significant loss of bacterial isolate while rinsing [36]. The dried slide was stained with crystal violet for 1 min and washed with indirect tap water. Subsequently, the slide was flooded with iodine for 1 min before being rinsed with tap water. A few drops of 95% ethanol were added to the slide for 5 s and washed with tap water. Following this, the inoculated slide was flooded with safranin and left for 45 s. The slide was washed with tap water and air-dried for a while. The cell shape and type of bacteria were observed using a light microscope with a magnification of 100×.

### 2.7. Biochemical Identification

#### 2.7.1. Hydrolysis of Enzymes

The isolated bacteria incubated overnight on marine agar were screened for the production of lipase, amylase, and protease enzymes using different types of agar composition. The lipase production of the bacterial isolate was quantified based on a spirit blue agar plate [37]; protease activity was tested using 1.0% (*w/v*) skimmed milk agar [38]; and amylase production was tested using 0.4% (*w/v*) starch agar [39], with some modifications. The agar (BD Difco^TM^, Thermofisher Scientific, USA) was prepared and sterilized at 121 °C for 20 min. For spirit blue agar, 3.0% (*v/v*) of olive oil was added to the sterilized media. A single colony of the bacteria isolate was streaked on an agar plate and incubated overnight at 37 °C to observe the halo zone. The incubated starch agar plate was flooded with iodine solution for 15–30 min prior to examining the halo zone. For all enzymes, the strength of enzyme activity was observed based on the appearance of the halo zone (diameter in mm) around the colony, following the method described by Dutta and Ghosh [40].

#### 2.7.2. Catalase Test

The catalase activity of the bacteria isolate was determined following the protocols described by Yang et al. [41]. A small amount of the bacterial colony was transferred to a glass slide using a sterile loop. A drop of 3% hydrogen peroxide (H_2_O_2_) was dropped on the glass slide and mixed. Bubble formation was observed within 5–10 s [42].

#### 2.7.3. Oxidase Test

Oxidase activity was detected with a commercial oxidase reagent (Becton, Dickinson and Company, Franklin Lakes, NJ, USA) according to the manufacturer’s instructions. Filter paper was saturated with freshly prepared oxidase reagent. The bacterial colony was smeared on the saturated filter paper. The development of a blue-purple color within 10 s was considered as a positive result [43].

#### 2.7.4. Susceptibility Test

The study of antibiotic sensitivity was tested following the work of Brown [44], with some modifications. The antibiotic disks (vancomycin, amoxycillin, ampicillin, streptomycin, kanamycin, penicillin G, and gentamicin) were placed over the laid bacteria isolate on nutrient agar plates. The plates were incubated at 37 °C overnight to measure the inhibition zone around the antibiotic disks.

#### 2.7.5. Determination of Marine Isolate

Reaction profiles for the biochemical tests were further analyzed using the BD BBLCrystal E/NF (Becton, Dickinson and Company, Franklin Lakes, NJ, USA) rapid test, as indicated by the manufacturer. The plate reaction was incubated at 37 °C for 24 h, as per the work of Popovic et al. [45].

### 2.8. Molecular Identification of Bacteria Isolate

The bacteria isolate from this experiment was identified through PCR amplification of the 16S rDNA gene, BLAST analysis, and comparison with sequences in the GenBank nucleotide database. The DNA of the selected marine bacteria was extracted using a QIAamp DNA microbiome kit (Cat. No.: 51704, QIAGEN GmbH, Hilden, Germany), following the procedures provided in the manufacturer’s protocol. The extracted DNA was used in 1% agarose gel electrophoresis and stained with ethidium bromide to visualize the bands [46]. The purified DNA was amplified with the universal primer 8F (5′- AGAGTTTGATCCTGGCTCAG-3′) [47] and 1492R (5′-TACGGTTACCTTGTTACGACTT-3′) [37]. Furthermore, 50 µL of the PCR reaction mixture containing MyTaq buffer, the DNA template, primer pair, and sterile distilled water was designated for the amplification program. The conditions for PCR were set as follows: initial denaturation at 95 °C for 1 min; 35 cycles of denaturation at 95 °C for 15 s; primer annealing at 55 °C for 15 s; elongation at 72 °C for 10 s, and final elongation at 72 °C for 4 min using the Master Cycler gradient (Eppendorf, Hamburg, Germany). The PCR product was sent to First BASE Sdn. Bhd. (Malaysia) for the sequencing. The analyzed DNA sequence was aligned and compared with the database in GenBank using BLAST, and the bacteria strain was identified as the strain with the nearest sequence similarity [48]. A dataset of potential orthologs was prepared by considering the database sequences that had > 98% sequence identity with the query sequence RLimb. The phylogenetic analysis was performed using the neighbor-joining method. Afterward, the 16S rDNA sequence of the bacterial strain RLimb was submitted to GenBank to obtain the accession number.

### 2.9. Statistical Analysis

The data were expressed as mean ± standard deviation (SD). Statistical analysis of the data was performed using GraphPad Prism software version 8.0 (GraphPad Software, San Diego, CA, USA). The differences between the groups were analyzed using a one-way analysis of variance (ANOVA), followed by Tukey’s post hoc test. Statistical significance was defined when the *p*-value was less than 0.05 (*p* < 0.05) [30,31].

## 3. Results and Discussion

### 3.1. Antibacterial Assay

In the present study, the bacteria culture extract was extracted from the cell-free supernatant (CFS) of the bacterial culture. These marine bacteria isolates were cultured from the culture collections at the Institute of Biotechnology Marine (IMB), Universiti Malaysia Terengganu (UMT), Terengganu, Malaysia. These bacteria isolates were isolated from the marine environment and selected given their ability to secrete bioactive compounds [49]. The bioactive compounds from the bacterial isolates were extracted using ethyl acetate and subjected to the current study. The results of the antibacterial activity of the bacteria culture extract using the disk diffusion method are shown in Table 1. The samples tested for this assay were the CFS of bacteria isolates and the solvent extract of the bacteria culture. The disks that were soaked with methanol were referred to as the negative control, while standard antibiotic disks were used as a positive control for this study. From Table 1, it can be observed that the zone of inhibition against four different types of test strains, namely *Bacillus cereus, Streptococcus uberis, Pseudomonas* sp., and *Vibrio parahaemolyticus*, showed that CFS exhibited no activity against these bacteria. No activity, or being inactive in an antibacterial assay, means that the sample cannot inhibit the growth of the test strains. In contrast, the sample that underwent solvent (ethyl acetate) extraction exhibited activity in this assay, indicating that it could inhibit the growth of the four test strains. The negative control disk showed no activity against the test strains, which indicates that methanol itself cannot inhibit the growth of test strains. The sample was moderately active against *B. cereus*, while it was significantly active against the other three test strains (*S. uberis, Pseudomonas* sp., and *V. parahaemolyticus*). The CFS prepared from the culture did not exhibit any activity due to the diluted nature of the compounds from the bacteria, while the solvent extract inhibited the growth of the test strains with the help of the solvent in the extraction of antibacterial compounds [50]. Employing organic solvents to extract the antibacterial compounds produced by bacteria can be helpful, as stated by Srilekha et al. [51]. Other studies have reported that the extraction of seaweeds using chloroform and ethyl acetate exhibited good activity in antibacterial tests [52,53]. This shows that using an organic solvent for extraction can separate the bioactive compounds from the raw sample. The results also showed that the sample extracted from the bacteria had a broad spectrum of antibacterial activity, as it inhibited the growth of both Gram-positive and Gram-negative test strains. A previous report showed that the antibacterial compound cyclo-L-proline-L-methionine was isolated from the marine bacteria *P. aeruginosa* obtained from the Antarctic sponge *Isodictya setifera* [54]. The secondary metabolites isolated from *P. aeruginosa* exhibited the maximum antibacterial activity, as reported by Palanichamy and Subramaniam [20]. Furthermore, according to Paul and Sinha [55], *P. aeruginosa* KUPSB12 exhibited antibacterial properties against six tested pathogenic bacteria. The production of antimicrobial activity can improve the specificity of the activity toward the biofilm of targeted bacteria in response to biofilm cultivation [56]. The sample was further tested for antifouling activities in the laboratory and aquarium.

### 3.2. Antifouling Activity

**Figure 2:**
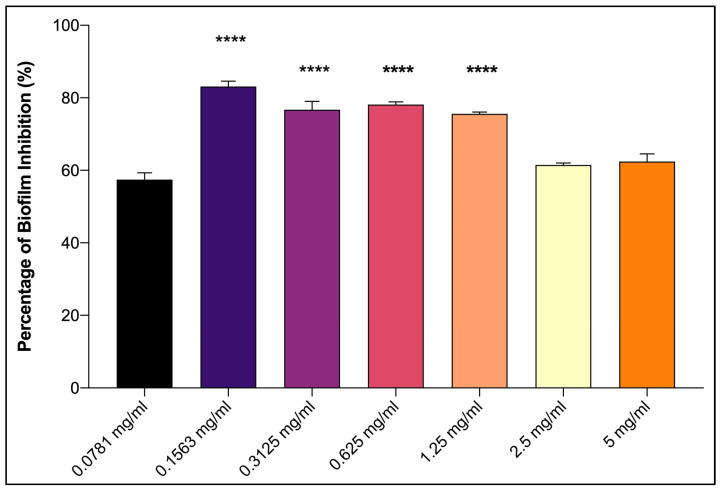
Antifouling activity of the bacteria culture extract against *P. aeruginosa* at various concentrations (0.0781 mg/mL–5 mg/mL). Data were presented as mean ± SD (n = 3). **** *p* < 0.0001 vs. lowest concentration (0.0781 mg/mL).

The assessment of antifouling can be conducted in a laboratory using the following two methods: the crystal violet assay and an aquarium with a mimicking environment. The crystal violet assay indicated antifouling performance by inhibiting the growth of the biofilm-forming bacteria *P. aeruginosa* [58]. *P. aeruginosa* is considered as the best biofilm-forming bacterial species because it can produce extracellular polymeric substances (EPS), which have approximately 40% of the dry weight of neutral polysaccharides [59]. The biofilm formed by the bacteria consists of 2–5% of bacterial cells, as well as EPS. According to Sarala et al. [60], *Pseudomonas* sp. is frequently isolated from biofouling samples, indicating that this species has the ability to form a biofilm. For this assay, the bacteria culture extract was diluted with dimethyl sulfoxide (DMSO) at various concentrations prior to serial dilution in a 96-well microtiter plate. The sample showed that the percentage of biofilm inhibition was higher at lower concentrations compared to higher concentrations (Figure 2). It inhibited the biofilm formation in the range of 61–83%, with the highest percentage of biofilm inhibition at a concentration of 0.1563 mg/mL. The concentration of bacteria culture extract that is lower than 0.1563 mg/mL will decrease the activity in biofilm inhibition. Thus, the concentration at 0.1563 mg/mL is an optimum concentration to inhibit biofilm formation. A concentration higher than 1.25 mg/mL showed a decreasing ability to inhibit the biofilm formation. The higher concentration of the bacteria culture extract may result in the attachment to the wall of the 96-well microtiter due to the concentrated sample and give a false positive result. A previous study by Chebbi et al. [61] also showed the same trend of biofilm inhibition activities of rhamnolipid RHW10 against biofouling strains. The spectrophotometric method used to quantify biofilm inhibition is simple and quick [34]. The absorbance values of the crystal violet serve as an indicator of the total attached biofilm at the bottom of the microtiter plate. The OD of the sterile medium with crystal violet dye was measured and deducted to account for the background absorbance [1]. This sample can be quantified as a good producer of antifouling compounds because it can inhibit the biofilm of *P. aeruginosa* at the lowest concentration, with a slight difference in the percentage of inhibition. It showed that the concentration of the bacteria culture extract did not affect the ability to inhibit the formation of the bacteria biofilm. The activity of antifouling is considered to be significant if ≥40% of biofilm inhibition was observed, while the activity below 0% indicates the promotion of biofilm formation [30]. The antifouling activities that employ this method show a significant reduction in biofilm formation due to the bacterial culture extract.

In this assay, acetic acid (resolubilizer) was added prior to gauging the absorbance to indirectly measure the biofilm attached to both the bottom and the wall of the wells [62]. Moreover, in this study, the M63 medium was used as the minimal medium to culture *P. aeruginosa*. A small amount of casamino acids and glucose in the medium formulation can most effectively promote the biofilm formation of all the test strains [63]. Thus, this medium can increase the efficiency of biofilm attachment on the microtiter plate.

**Figure 3:**
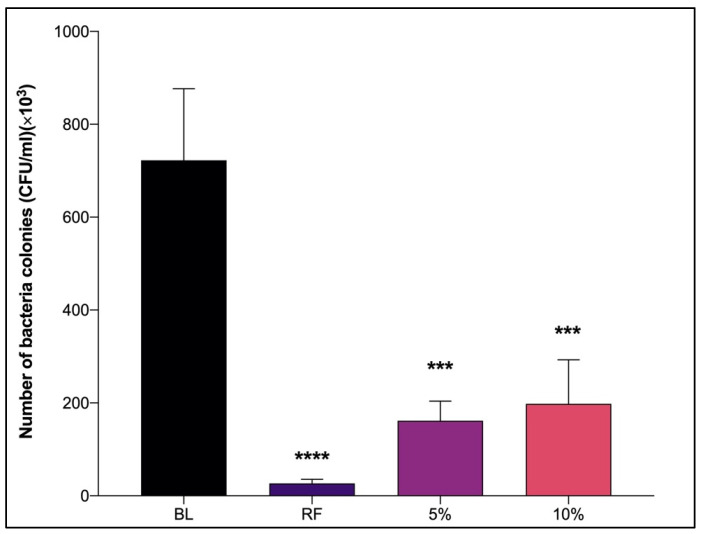
The number of biofilm-forming bacteria colonies on the painted panels at two different concentrations; 5% and 10% (*w/v*) of bacteria culture extract. Data were presented as mean ± SD (*n* = 3). BL: Panel coated with blank (control); RF: Panel coated with commercial antifouling paint. *** *p* < 0.001, **** *p* < 0.0001 vs. BL group.

The fouling process involves the following three main stages: the formation of the conditioning film, microfouling settlement, and macrofouling attachment [64]. This experiment, which took place in an aquarium, involved the second phase of the fouling process, the formation of microfouling (bacteria). The beginning of the second phase was triggered by the combination of the conditioning film in which the organic molecules in the water attached to the immersed surface and the dead cells from phase one, as stated by Zulkifli et al. [13]. Another antifouling method is spray painting bacteria culture extract samples onto the panels and hanging them in the aquarium with seawater. The tank was supplemented with sterile artificial seawater as an additional nutrient and placed on a motor to create waves to mimic the real environment. The panels were retrieved each day for 3 days of the experiment. The panels were retrieved from the aquarium within 24 h of the experiment, and subsequently subjected to colony number counting. This interval was sufficient for the simulation test of antifouling activities in this study because the biofilm produced by bacteria developed within minutes to hours after immersion. Bacteria are considered to be pioneering organisms, namely the first organism to settle on surfaces [65]. The panels were then swabbed using a sterile cotton swap and spread on Zobell marine agar (ZMA). As shown in Figure 3, the number of colonies of bacteria on the panels was counted using a colony counter. The number of biofilm-forming bacteria colonies that adhered to the panel coated with only primer paint was discovered to be 722.5 × 10^3^ CFU/mL and significantly reduced to 198 × 10^3^ CFU/mL on the panel at a concentration of 10%. Similarly, the panel at a concentration of 5% significantly reduced the number of biofilm-forming bacteria colonies to 161.8 × 10^3^ CFU/mL. This result showed that the bacteria culture extracts at lower concentrations are more effective at inhibiting the adhesion of biofilm-forming bacteria on panels compared to higher concentrations with a slightly different number of colonies. These results showed that both concentrations of bacteria culture extracts can inhibit the biofilm formation on the surface of panels by having fewer colonies. Normally, the higher the concentration of the sample, the higher the percentage inhibition of biofilm formation. It is because the higher concentration of sample specifies the larger amount of bacteria culture extract and is expected to result in maximum activities. However, the lower concentration of bacteria culture extract demonstrated better inhibition of biofilm formation in this study. It is due to the fact that higher concentration of bacteria culture extract may promote the adhesion of bacteria and increase the number of colonies of biofilm-forming bacteria. An increase in the biofilm and bacterial growth can be observed in some extracts and concentrations. This might be because the chemicals in the extracts have enriched the culture media [66]. Other studies also noted this pattern [67,68]. When there was a decrease in bacterial density in planktonic and biofilm formation but an increase in biomass, the reason for the increase might be the overproduction of the matrix and/or a change in bacterial shape while in contact with the extract media [69]. The result for the aquarium test was equivalent to the antifouling test using a 96-well microtiter plate. The results in both tests showed that the lower concentration of bacteria culture extract can more effectively inhibit the formation of bacteria biofilms than at higher concentration. This indicated that the sample (bacteria culture extract) was good at producing antifouling compounds to prevent the attachment of the biofilm-forming bacteria. Therefore, it can be concluded that this bacteria culture extract can be considered as a substitution for a toxic anti-fouling agent because it can inhibit biofilm formation by biofilm-forming bacteria even in a small amount. It is a good strategy to find a substitute sample that exhibits the same activities using fewer samples for commercialization purposes.

### 3.3. Characterization of Bacteria Isolate

**Figure 4:**
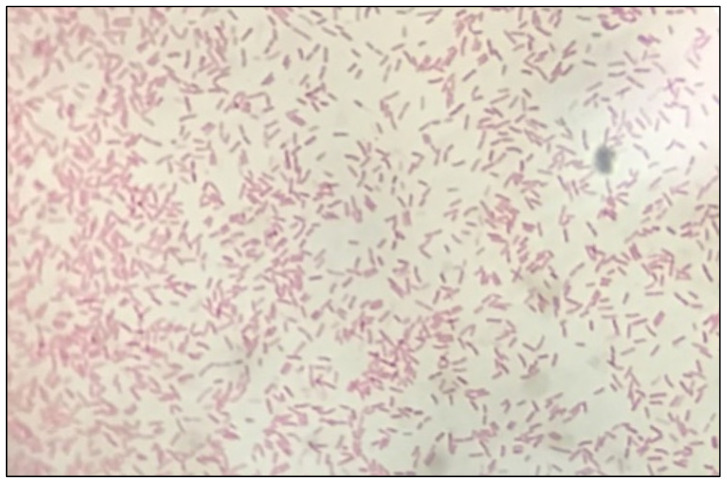
Microscopic observation of isolate RLimb indicated rod-shape and Gram-negative bacteria (magnification at 100×).

The morphology of bacteria colonies and biochemical characterization can help in the partial identification of marine isolates. The morphological and colonial characteristics of the isolate, as shown in Table 2, were obtained when it was cultured on marine agar and observed under a light microscope. The isolate RLimb showed a rod shape and microscopically, it was a Gram-negative bacteria (Figure 4). The isolate was sensitive to experimental doses of streptomycin and gentamicin, as shown in Table 3. However, the isolate was resistant to vancomycin, penicillin G, amoxicillin, ampicillin, and kanamycin. The sensitivity test of the isolate RLimb was shown by the clear halo zone around the antibiotic disks. According to the study by Ahmadi et al. [70], 74.41% of *P. aeruginosa* in their study were resistant to vancomycin. *P. aeruginosa* shares characteristics with other Gram-negative pathogens, such as an outer polysaccharide layer, multi-drug efflux transportees, and a high level of biofilm formation, which collectively increase the probability of resistance toward antibiotics [71].

**Figure 5:**
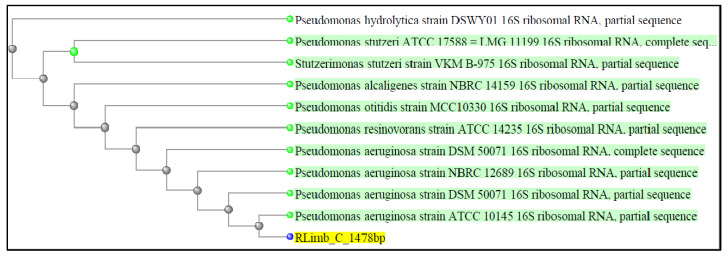
Phylogenetic tree construction of isolated bacteria strains *P. aeruginosa*.

Several biochemical tests were performed to characterize the marine bacterial isolate. The biochemical characteristics of the isolate are specified in Table 4 and Table 5. The results in both tables were analyzed and determined using the BBLCrystal E/NF plate test. The biochemical activities of the bacteria culture extract were determined by the color change of the chemical solution in the plate test. The isolate produced lipase and protease enzymes, but not amylase enzymes. The production of lipase, protease, and amylase was indicated by the clear inhibition zone around the isolate [37]. Furthermore, the isolate exhibited catalase and oxidase activity by producing air bubbles and turning blue. In the catalase test, the isolate produced free oxygen in the form of air bubbles as soon as hydrogen peroxide was added [38]. Whether it was oxidase positive was determined by the oxidation of the Kovacs reagent, which turned blue [72]. Kumar et al. [73] stated that *P. aeruginosa* showed maximum protease activity in different media. In another study by Paul and Sinha [55], *Pseudomonas* sp. KUPSB12 was indicated to hydrolyze catalase and protease enzymes but not amylase enzymes. All of the characteristics shown in this study point to the phenotype characteristics of the genus *Pseudomonas.* To identify the isolated bacteria, 16S rDNA gene sequencing was performed. The analysis of marine bacterial isolates was performed via basic local alignment search tool (BLAST) analysis for the 16S rDNA sequence obtained using the NCBI GenBank database by showing sequence similarity. This revealed that the sequence had a sequence similarity of 100% to the *P. aeruginosa* strain DSM 50071 in the NCBI database. The FASTA sequence was then submitted to GenBank for the accession number. The phylogenetic tree was constructed using the neighbor-joining method to analyze the most similar sequence, as shown in Figure 5. The phylogenetic tree indicated that the isolated bacteria had a close relation to *P. aeruginosa*. Therefore, the isolated bacteria strain was assigned as the *P. aeruginosa* strain RLimb (accession number: OP522351).

## 4. Conclusions

Through the crystal violet assay and aquarium test, the bacterial isolate was demonstrated to produce antifouling compounds. This isolate also exhibited activities in the antibacterial test that inhibited the growth of the test strains. Exploring the relationship between antifouling activity and antibacterial activities is highly recommended to screen all the isolates that have the potential to reduce the biofilm of a test strain in a laboratory test. The isolate, the *P. aeruginosa* strain Rlimb, was demonstrated via isolation, characterization, and identification to be a substitute producer of antifouling compounds; furthermore, it does not harm the environment, as it is naturally isolated from marine bacteria. It is defined as harmless to the environment because it does not use toxic chemicals to prevent the formation of biofilms. This isolate lends credence to the current study regarding the production of compounds with the highest volume, with low costs and environmentally friendly materials. It can be used in the industry for paint production to prevent the early-stage formation of foulers.

## Figures and Tables

**Table 1:** Antibacterial activities of the bacteria culture extract against *B. cereus*, *S. uberis*, *P. aeruginosa*, and *V. parahaemolyticus*.

Test Strains Samples	*B. cereus*	*S. uberis*	*P. aeruginosa*	*V. parahaemolyticus*
Cell-free supernatant of bacteria isolates	−	−	−	−
Ethyl acetate extract of bacteria isolates	++	+++	+++	+++
Antibiotic				
Kanamycin (30 μg/disc)	++++	++++	NA	NA
Gentamicin (10 μg/disc)	NA	NA	++++	++++
Methanol-soaked disk	−	−	−	−

NA: Not available; (−): zone of inhibition (inactive); (+): 7–8 mm zone of inhibition (mildly active); (++): 8–9 mm zone of inhibition (moderately active); (+++): 9–11 mm zone of inhibition (significantly active); (++++): 11-13 mm zone of inhibition (strongly active) [57].

**Table 2:** Isolated bacteria based on morphology and colony characteristics.

Isolate	Rlimb
Morphological characteristics	
Gram reaction	Gram-negative
Size and shape	Long rod-shape bacteria
Colonial characteristics	
Form	Circular
Colony size	Intermediate
Margin	Entire
Pigmentation	No pigmentation
Elevation	Flat
Opacity	Opaque

**Table 3:** Antibiotic sensitivity tests of isolate RLimb against recommended doses of antibiotic.

Antibiotic Used	Results
Vancomycin (5 μg/disc)	−
Penicillin G (10 U/disc)	−
Amoxicillin (25 μg/disc)	−
Streptomycin (10 μg/disc)	+
Ampicillin (10 μg/disc)	−
Kanamycin (30 μg/disc)	−
Gentamicin (10 μg/disc)	++

(−): no zone of inhibition (inactive); (+): 7–9 mm zone of inhibition (mildly active); (++): 10–15 mm zone of inhibition (moderately active).

**Table 4:** Carbohydrate fermentation properties of isolate RLimb.

Carbon Source	Results
Arabinose	−
Mannose	−
Sucrose	−
Melibiose	−
Rhamnose	−
Sorbitol	−
Mannitol	−
Adonitol	−
Galactose	−
Inositol	−

(−): Not available.

**Table 5:** Biochemical characterization of isolate RLimb.

Tests	Results
Catalase	+
Oxidase	+
Protease	+
Lipase	+
Amylase	−
p-nitrophenyl phosphate	+
p-nitrophenyl *α*-*β*-glucoside	−
p-nitrophenyl *β*-galactoside	−
Proline nitroanilide	+
p-nitrophenyl bis-phosphate	−
p-nitrophenyl xyloside	−
p-nitrophenyl *α*-arabinoside	−
p-nitrophenyl phosphorylcholine	+
p-nitrophenyl *β*-glucuronide	−
p-nitrophenyl-N-acetyl glucosaminide	−
*γ*-L-glutamyl p-nitroanilide	+
Esculin hydrolysis	−
Phenylalanine deamination	+
Urea hydrolysis	+
Glycine degradation	+
Citrate utilization	+
Malonate utilization	+
Tetrazolium reduction	−
Arginine catabolism	+
Lysine catabolism	+

(+): Available; (−): Not available.

## Data Availability

Data is available upon request.
